# Seasonal Dynamics of Macroinvertebrate Communities in Offshore Mussel Aquaculture in the Southern Black Sea: Implications for Diversity

**DOI:** 10.3390/life15091471

**Published:** 2025-09-19

**Authors:** Eylem Aydemir Çil

**Affiliations:** Department of Environmental Engineering, Faculty of Engineering and Architecture, Sinop University, 57000 Sinop, Türkiye; eylemaydemir@sinop.edu.tr

**Keywords:** mussel aquaculture, macroinvertebrates, *Jassa marmorata*, artificial reefs, benthic diversity, ecological indicators

## Abstract

This study investigates the taxon composition, seasonal variations, and diversity dynamics of macroinvertebrate communities associated with *Mytilus galloprovincialis* cultivated in mussel longline systems in the central Black Sea. Monthly sampling conducted between September 2023 and August 2024 yielded a total of 99,719 individuals representing 20 taxa. The communities were predominantly dominated by amphipods, particularly *Jassa marmorata* (71%) and *Stenothoe monoculoides* (28%). Individual abundance peaked in autumn, whereas taxon richness reached its highest levels in summer. ANOVA results revealed significant seasonal differences in diversity indices (*p* < 0.05). Multivariate analyses, including NMDS and RDA (PCA proxy), indicated distinct seasonal clustering, with pH (41%) and salinity (35 g/kg) identified as the primary environmental drivers of community composition. These findings demonstrate that offshore mussel longline systems function not only as aquaculture infrastructure but also as reef-like artificial habitats that support benthic diversity. The dominance of opportunistic and detritivorous amphipods, along with their sensitivity to environmental gradients, suggests their potential utility as bioindicators for ecological monitoring.

## 1. Introduction

When compared with other semi-enclosed seas such as the Mediterranean and the Baltic, the Black Sea is distinguished by its moderate salinity and the presence of a permanent anoxic layer in its deep waters, whereas the Mediterranean is characterized by high salinity and well-oxygenated conditions and the Baltic by very low salinity and limited taxon diversity [1,2]. This unique hydrographic structure directly shapes the region’s physical, chemical, and biological characteristics. Surface waters are dominated by freshwater inputs from rivers, resulting in salinity levels as low as approximately 17 g/kg. With increasing depth, more saline, Mediterranean-origin waters occupy the deeper layers, leading to the formation of a pronounced halocline [1,2]. This density stratification severely restricts vertical mixing between surface and bottom waters, rendering the layers below 150–200 m anoxic. Beneath this anoxic zone, hydrogen sulfide (H_2_S) accumulates, making the Black Sea one of the rare marine systems where aerobic life is absent in the deep-water column [3].

Such stratification directly influences phytoplankton productivity, zooplankton composition, and benthic life. In addition, high nutrient inputs—particularly nitrogen and phosphorus—delivered by rivers trigger eutrophication processes, leading to algal blooms, oxygen depletion, and an overall shaping of the ecosystem [4]. These dynamics significantly shape the distribution and diversity of macrozoobenthic communities in the region.

The Black Sea, with its rich biological diversity and ecological heterogeneity, holds significant importance at both regional and global scales. Its benthic ecosystems, characterized by diverse habitats and high taxon richness for some ecosystems, play a critical role in maintaining ecological balance and sustaining biological productivity [5,6,7]. In the mediolittoral and infralittoral zones of the Black Sea and the Sea of Marmara, *M. galloprovincialis* forms dense beds on hard substrates [3,8,9,10,11,12].

Mussel aquaculture systems are valuable not only as units of food production but also in terms of the ecosystem services they provide, including habitat provision, contribution to nutrient cycling, and an increase in taxon richness. In recent years, there has been growing interest in the role of these systems as reef-like structures [13,14].

Macroinvertebrates are widely recognized as effective bioindicators due to their sensitivity to environmental changes and their integral roles within benthic food webs [15,16]. In the Black Sea, *M. galloprovincialis* beds are generally observed in shallow waters, but they can extend to depths of 30–50 m, and in some areas, even down to 80 m [17,18]. These mussels are capable of colonizing a wide variety of habitats, ranging from coastal hard substrates and rocky shores to artificial structures and deep-sea muds.

As suspension feeders, *M. galloprovincialis* consume phytoplankton, zooplankton, organic detritus, bacteria, and dissolved organic matter present in the water column [19,20,21]. The productivity of natural populations can be limited by factors such as physiological stress, food scarcity, predation, and density-dependent pressures [22,23]. Macrobenthic organisms associated with mussel beds are generally categorized into ecological groups such as epibenthic fauna, epiphytic fauna, infauna, and free-living fauna [24,25]. The composition of these groups is influenced by environmental conditions and by the accumulation of particulate organic matter within the mussel beds [26].

In recent years, numerous scientific studies have been conducted in the Black Sea focusing on the monitoring and conservation of benthic invertebrates (e.g., Oligochaeta, Chironomidae larvae). These efforts provide valuable insights into ecosystem health assessment and diversity conservation. Benthic invertebrates are indispensable components for maintaining ecosystem balance and water quality, and their protection is crucial for ensuring the region’s long-term ecological sustainability [27].

The Black Sea’s water temperature, ranging from 7–25 °C, and salinity levels, ranging from 17–20 g/kg, together offer favorable conditions for mussel aquaculture [28,29]. Mussel farms in the region are designed as surface-deployed longline systems, based on the expectation that the high phytoplankton concentrations in surface waters will promote mussel growth. Studies on macrobenthic invertebrates associated with *M. galloprovincialis* have identified amphipods as one of the dominant groups inhabiting these environments [30]. Amphipods utilize mussel beds both as shelter and as feeding grounds within these detritus- and microorganism-rich habitats. Taxa belonging to the families Gammaridae and Caprellidae make significant contributions to the ecological richness of mussel beds. Such mutualistic associations increase the taxon richness and structural complexity of mussel assemblages, thereby supporting ecosystem sustainability.

This study posits that the structure and diversity of macroinvertebrate communities in offshore mussel aquaculture systems are strongly influenced by environmental gradients—particularly pH and salinity—and that under moderate environmental stress conditions, opportunistic amphipods (e.g., *J. marmorata*) tend to dominate. Accordingly, these communities may serve as effective biological indicators for assessing environmental quality.

## 2. Materials and Methods

### 2.1. Longline System

**Figure 1:**
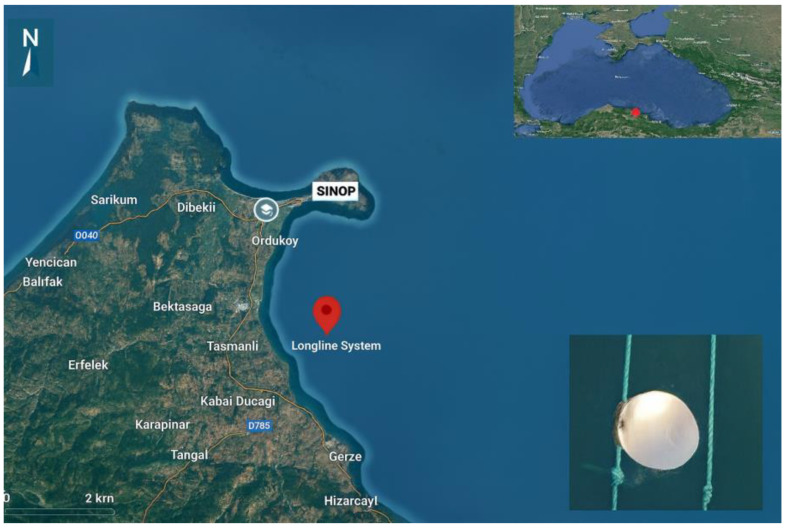
Coordinates of the longline system.

**Figure 2:**
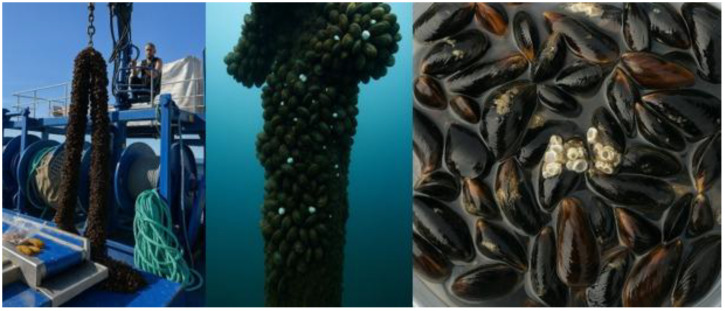
Longline system.

The facility is established within a primary aquaculture zone, officially designated as the most suitable area for aquaculture activities in Sinop, and consists of a surface-deployed longline system (Figure 1). The nearest point of the project site to the shore is distance corrected to approximately 4.5 km (Figure 1), with depths ranging from 35–42 m. The study was conducted in the central Black Sea, in offshore waters at depths of 25–27 m, between September 2023 and August 2024. This marine area was selected due to its reduced exposure to harsh weather conditions such as strong winds, currents, and high waves. The system was designed in accordance with local environmental conditions and consists of twelve submerged longline units, each measuring 8 m in length (Figure 2).

### 2.2. Environmental Parameters

Water temperature and salinity data were measured monthly between September 2023 and August 2024 using a YSI 6600 multi-parameter probe. Measurements were conducted in situ by immersing the probe directly into the water from the sampling vessel, and the data were recorded immediately. All water parameter data collected during the one-year sampling period were compiled into an Excel file and prepared for subsequent analyses.

### 2.3. Mussel Sample Collection

In the longline system, each rope was suspended within the water column using buoys and weights. Sampling was conducted over a 12-month period between 09:00 and 14:00 local time (Türkiye). On twelve standardized mussel ropes, uniform in length, thickness, and material, stopper pins were positioned at 90 cm intervals to prevent mussel slippage, and the mussels sampled were approximately 4–5 cm in size and 12–14 months old. Macroinvertebrate samples were collected from 30 cm segments located between these pins. To avoid resampling, sampled sections were marked with colored ropes. To ensure seasonal representativeness, three replicate samples were collected monthly from mussel ropes suspended at a depth of 3 m using standardized mesh bags.

Macroinvertebrate sampling was conducted using mesh bags measuring 30 cm in length, with a mesh size of 0.25 mm, equipped with Velcro fastenings on three sides. These bags were secured in situ around the designated rope segments. Mussels and associated organisms were gently loosened by hand into the bag. The Velcro closures were then carefully opened and resealed to remove the contents in a controlled manner [30].

Collected material was emptied into a container on board and transferred with a small scoop into plastic jars with a capacity of 500–1000 L. Samples were preserved in 96% ethanol for subsequent processing. The total number of individuals obtained from the three replicates was divided by three to calculate the mean individual abundance per unit.

Once transported to the laboratory, mussel samples were washed using a double-layered sieving system with mesh sizes of 2 mm and 0.5 mm. Organisms retained on the 0.5 mm sieve were included in the study, while smaller fractions were excluded. The retained material was sorted under a stereomicroscope with appropriate illumination, separated into taxonomic groups, labeled, and preserved in 75% ethanol for taxon identification. Each specimen was identified to the lowest possible taxonomic level. However, in cases where individuals were fragmented or lacked sufficient morphological features, taxa were classified at the genus level. Taxon lists and diversity index results were reported according to the sampling site.

For the identification of marine macrobenthic invertebrates, standard morphological keys and regional faunal references were used. For members of the class Polychaeta, Refs. [31,32] were consulted, while Refs. [27,33] served as primary references for mollusks. For Crustacea, particularly Amphipoda and Decapoda, Refs. [34,35] were used. Taxon identifications and current taxonomic information were further verified using digital databases such as the World Register of Marine Species [36] and the Ocean Biodiversity Information System [37].

### 2.4. Statistical Analyses and Diversity Indices

All statistical analyses were performed using R software (v4.3.1) with the vegan and ggplot2 packages. Prior to applying parametric tests, the assumptions of normality and homogeneity of variance were evaluated using the Shapiro–Wilk test and Levene’s test, respectively. Where assumptions were not met, the Kruskal–Wallis test was applied as a non-parametric alternative to one-way ANOVA.

Community composition patterns were assessed using Non-Metric Multidimensional Scaling (NMDS) based on the Bray–Curtis dissimilarity metric, which is widely used for ecological community data. To further test for differences in community composition among groups, Analysis of Similarities (ANOSIM) was performed using Bray–Curtis distances, providing an R statistic as a measure of group separation.

To explore the relationships between environmental variables and macroinvertebrate assemblages, Redundancy Analysis (RDA) was conducted. Prior to RDA, taxon abundance data were Hellinger-transformed to reduce the influence of highly abundant taxa. Environmental parameters (temperature, salinity, dissolved oxygen, and pH) were standardized, and forward selection with Monte Carlo permutation tests (999 permutations) was used to determine the significance of explanatory variables included in the final model.

Diversity and evenness metrics were calculated to characterize monthly macroinvertebrate communities using the mean values derived from three replicates. These included: the Shannon–Wiener Diversity Index (H′) [38]

H′ = −Σ (Pi × log_2_ Pi), i = 1 → S     where     Pi = Ni/N

where:

H′ = Shannon–Wiener Diversity Index
Pi = Proportion of individuals in taxon i
Ni = Number of individuals in taxon i
N = Total number of individuals in the sample
S = Total number of taxa in the sample (taxon richness)

This index ranges from 0 to 5, although it rarely exceeds 1; values closer to 5 indicate higher taxonomic diversity.

Pielou’s Evenness Diversity Index was calculated using the formula [39]:

E = H′/log_2_S or H′/Hmax

where:

S = Total number of taxa in the sample (taxon richness)
H′ = Shannon–Wiener Diversity Index

This index ranges from 0 to 1, with values approaching 1 indicating that taxa are distributed relatively evenly throughout the community. The most commonly used formula for Simpson’s Diversity Index is as follows:

D = Σ (ni/N)^2^, i = 1 → S

D = Simpson’s Diversity Index (measures the probability that two individuals randomly selected from a sample will belong to the same taxon)
S = Total number of taxa in the sample (taxon richness)
ni = Number of individuals in taxon i
N = Total number of individuals in the sample
ni/N = Proportional abundance of taxon i

where ni is the number of individuals in taxon i, and N is the total number of macrobenthic invertebrate individuals.

## 3. Results

### 3.1. Physicochemical Parameters and Seasonal Variations

**Figure 3:**
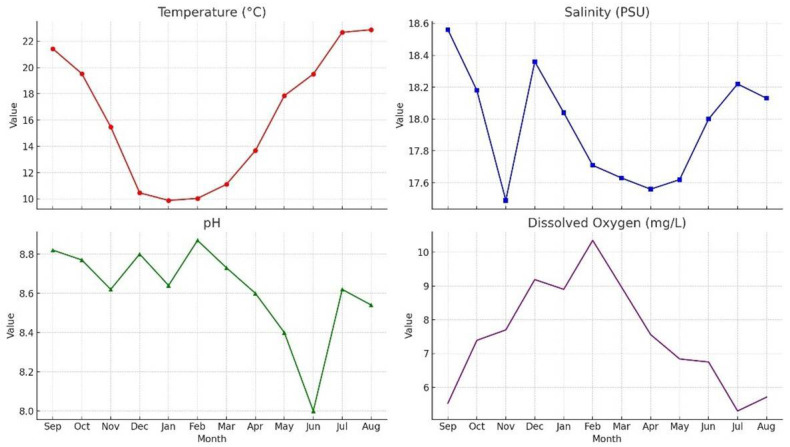
Monthly physicochemical parameter data.

An overview of the water parameters measured throughout the one-year sampling period is presented in Figure 3:

Water Temperature (T): Water temperature exhibited a typical seasonal cycle, with the lowest value recorded in winter (9.88 °C in January) and the highest in summer (22.88 °C in August). The lowest temperatures occurred in January and February, followed by a gradual increase starting in May.

Salinity (S): Salinity fluctuated within a relatively narrow range throughout the year, reaching its minimum in autumn and maximum at the end of summer. These variations are likely influenced by environmental factors such as freshwater inputs and evaporation rates.

pH: pH values remained within a slightly alkaline range (8.00–8.87). Increases during winter may be associated with lower temperatures and higher dissolved oxygen levels, whereas decreases observed in summer could be linked to increased biological activity and the decomposition of organic matter.

Dissolved Oxygen (DO): Dissolved oxygen levels peaked in February at 10.35 mg/L and reached their lowest level in July at 5.30 mg/L. This pattern corresponds to the reduced solubility of oxygen at higher temperatures and increased biological oxygen demand during the warmer months.

### 3.2. Macroinvertebrate Community

During the 12-month sampling period conducted within the mussel longline system in the Black Sea, a total of 99,719 macroinvertebrate individuals representing 20 taxa were recorded (Table 1). The identified taxa encompassed a wide range of invertebrate groups, including Crustacea, Mollusca, Polychaeta, Cirripedia, Platyhelminthes, Cnidaria, and Nematoda. This diversity reflects both the ecological heterogeneity and the structural complexity of the benthic community associated with mussel aquaculture (Table 1).

### 3.3. Taxon Distribution and Seasonal Variations

Monthly taxon distribution exhibited pronounced temporal (seasonal) variability, largely shaped by the dominance of *J. marmorata*, which accounted for approximately 71% of the total individuals (Table 1). It should be noted, however, that all of these dominant taxa in Table 1 are considered to play key roles in the trophic structure and habitat dynamics of benthic environments associated with mussel beds.

**Figure 4:**
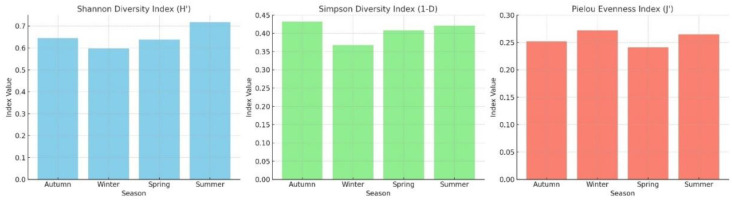
Shannon–Wiener (H′), Simpson’s (1–D), and Pielou’s Evenness (J′) diversity indices.

ANOVA results indicated statistically significant seasonal differences in the Shannon–Wiener Diversity Index (*p* < 0.05), with the highest diversity observed during summer and the lowest values recorded in winter. To evaluate the ecological diversity of the macrozoobenthic community structure, the Shannon–Wiener (H′), Simpson’s (1–D), and Pielou’s Evenness (J′) diversity indices were calculated. The index values revealed notable temporal variations in diversity and distribution patterns over the 12-month study period (Figure 4).

Although the Kruskal–Wallis test revealed no statistically significant differences (*p* > 0.05), taxon richness peaked in summer, while individual abundance reached its maximum in autumn. This pattern suggests asynchronous responses of the community to environmental variables.

**Figure 5:**
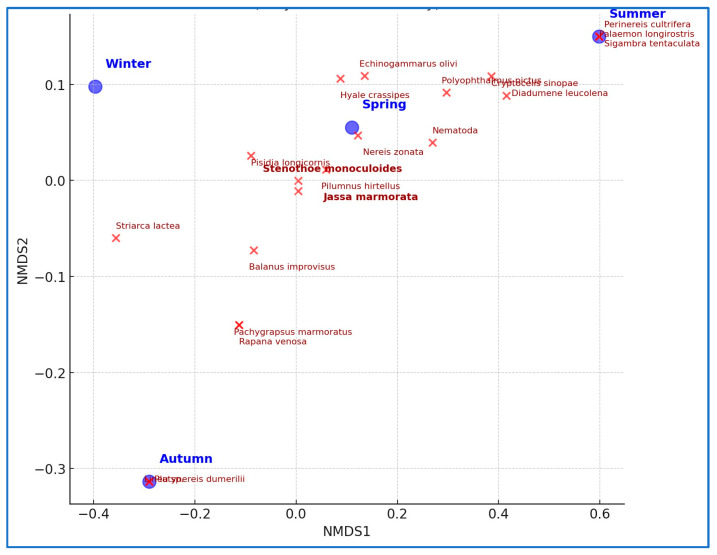
NMDS ordination of macroinvertebrate communities showing seasonal clustering based on Bray–Curtis dissimilarity.

The results of NMDS analysis, based on the Bray–Curtis dissimilarity of Hellinger-transformed taxon abundance data, revealed distinct seasonal clustering of macroinvertebrate communities. The greatest separation occurred between summer and autumn assemblages, while spring and autumn samples exhibited the highest similarity. Although the Kruskal–Wallis test indicated no statistically significant differences (*p* > 0.05), taxon richness was highest in summer, whereas individual abundance peaked in autumn. Notably, *J. marmorata* and *S. monoculoides* were strongly associated with spring assemblages, *D. leucolena* dominated summer samples, and *N. zonata* was more evenly distributed across seasons. This pattern suggests asynchronous responses of dominant taxa to seasonal environmental variations (Figure 5).

**Figure 6:**
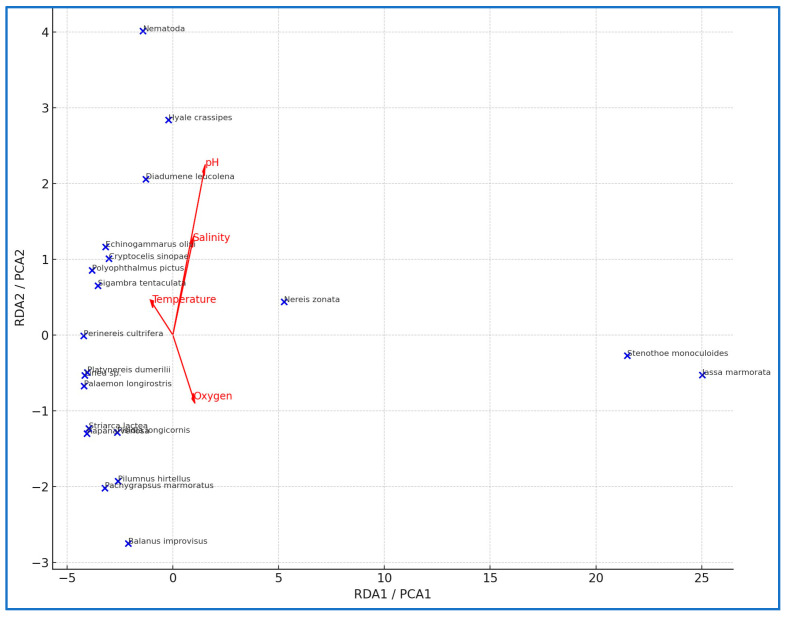
Taxon–environment relationships based on the RDA (PCA proxy) biplot.

A pronounced increase in both taxon richness and individual abundance was observed during summer and early autumn (June–September). This seasonal peak coincides with periods of higher temperatures and reduced hydrodynamic impact, indicating enhanced macrofaunal productivity. The consistent dominance of *J. marmorata* during these months reflects the taxon’s rapid colonization ability and competitive advantage on artificial structures. In contrast, the lower and more stable abundances recorded in winter and spring are consistent with suppressed benthic activity under low temperatures and elevated oxygen conditions (Figure 6).

Total abundance reached its highest levels in autumn, potentially reflecting a post-reproductive dispersal phase of dominant taxa following their summer breeding period. Conversely, winter was characterized by a notable decline in both richness and abundance, likely driven by stressors such as low temperatures, high dissolved oxygen levels, and limited organic matter availability.

**Figure 7:**
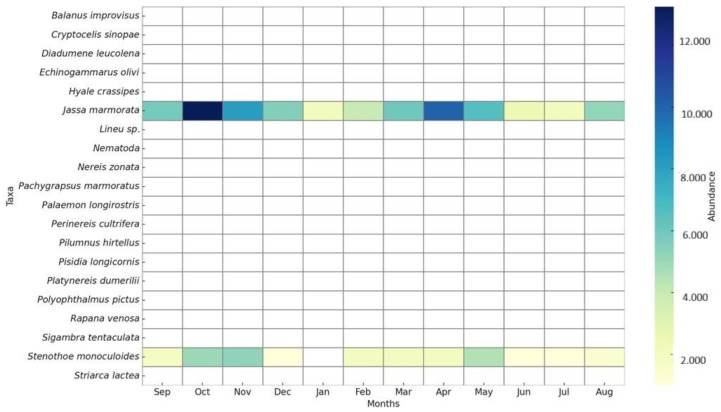
Shaded bar graphs showing the monthly macrofaunal composition recorded in the longline system.

While the dominant amphipods *J. marmorata* and *S. monoculoides* largely shaped the overall temporal trends, several low-abundance taxa such as *Balanus improvisus*, *C. sinopae*, *H. crassipes*, and *N. zonata* contributed to short-term peaks in richness during specific sampling periods (e.g., S4, S9) (Table 2). Although these episodic fluctuations had minimal influence on total abundance, they underscore the ecological variability of the community and the occurrence of sporadic settlement events (Figure 7).

The RDA biplot illustrated the relationships between taxa and environmental variables (pH, dissolved oxygen, temperature, and salinity) (Figure 6). These findings are of considerable importance for understanding the influence of environmental variables on taxon distribution and habitat preferences.

The results of RDA indicated that pH and temperature were the most influential physicochemical factors shaping taxa distribution. pH showed a strong relationship to sensitive taxa such as *H. crassipes, D. leucolena*, and *C. sinopae*, suggesting that even small fluctuations in alkalinity may regulate their settlement and persistence. In contrast, the dominant amphipods *J. marmorata* and *S. monoculoides* were primarily aligned with temperature along the first axis, reflecting their seasonal proliferation during warmer months. The opportunistic polychaete *N. zonata* was more closely associated with oxygen, showing higher abundances during winter. Rare or episodic taxa (*Rapana venosa*, *Platynereis dumerilii*, *Striarca lactea*) exhibited no strong correlation with environmental gradients, instead reflecting sporadic recruitment events. Overall, the results demonstrate a clear seasonal structuring of the community, with pH influencing sensitive taxa, temperature driving dominant taxa, and oxygen regulating opportunistic taxa (Figure 6). This pattern indicates that community structure beneath mussel aquaculture is strongly influenced by seasonal hydrographic variations.

Overall, the mussel longline macrofaunal communities exhibited strong seasonality, driven primarily by a few opportunistic and structurally dominant taxa. Taxon richness and abundance were not evenly distributed throughout the year but instead followed distinct seasonal patterns shaped by environmental conditions and the life-history strategies of constituent taxa.

These findings support the hypothesis that: “Offshore longline mussel aquaculture systems create reef-like structures that promote seasonally variable yet generally increased taxon richness, evenness, and biomass within macroinvertebrate communities. These structures also influence the distribution of taxa—particularly amphipods and mollusks—that are sensitive to changes in environmental variables such as temperature, pH, and oxygen”.

### 3.4. Abundance and Taxon Richness

**Figure 8:**
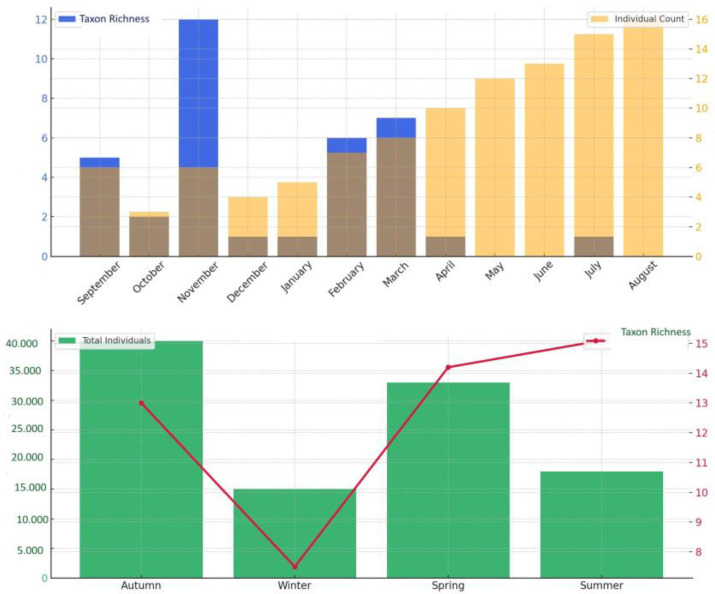
Monthly and seasonal distribution of taxon abundance.

Macrofaunal individual abundance peaked in autumn, whereas the lowest values were recorded during summer. In contrast, taxon richness reached its maximum in summer, with a total of 15 taxa identified during this period (Figure 8).

The monthly and seasonal patterns of taxon richness and individual abundance (Figure 8) clearly reveal the temporal dynamics of macrofaunal communities in the investigated offshore mussel aquaculture system. These patterns appear to be shaped by both seasonal variations in environmental conditions and the life-cycle characteristics of the taxa present.

**Figure 9:**
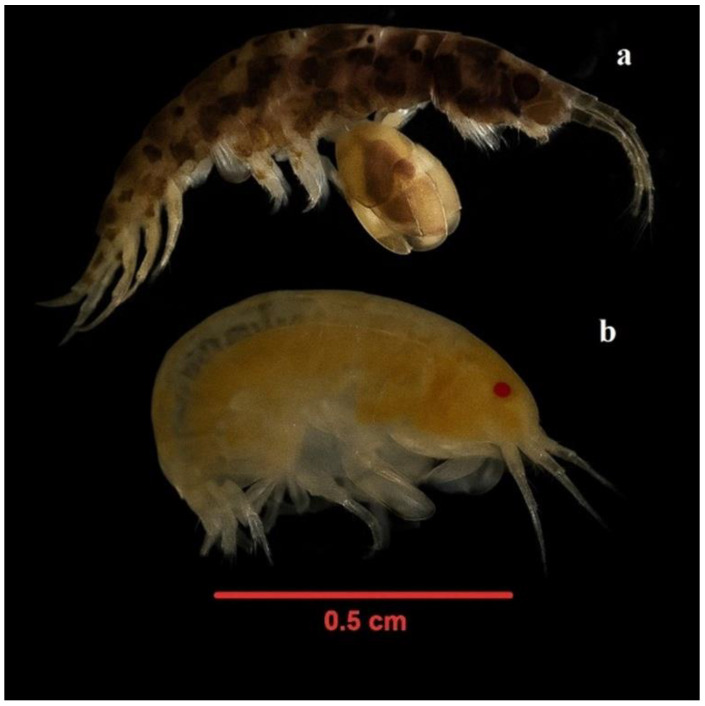
Image showing *J. marmorata* Holmes, 1905 (**a**) and *S. monoculoides* (Montagu, 1813) (**b**).

Total abundance reached its peak in autumn, potentially reflecting a dispersal phase of dominant taxa following their summer reproductive period (Figure 8). In contrast, winter was characterized by a marked decline in both richness and abundance, primarily attributable to stress factors such as low temperatures and limited organic matter availability. Elevated dissolved oxygen concentrations observed during this season are instead interpreted as a natural consequence of reduced biological activity rather than a stressor. The dominant amphipods *J. marmorata* and *S. monoculoides* (Figure 9) largely shaped the overall temporal trends, while several low-abundance taxa such as *B. improvisus*, *C. sinopae*, *H. crassipes*, and *N. zonata* contributed to short-term increases in richness during specific sampling periods (e.g., S4, S9). Eurytopic amphipods such as *Jassa*, *Corophium*, and *Gammarus tigrinus* exhibit broad tolerance to fluctuations in temperature, salinity, and pH, enabling them to thrive under diverse and often stressed environmental conditions [39]. Although these transient fluctuations had little effect on total abundance, they highlight the ecological variability of the community and the occurrence of episodic settlement events.

In conclusion, offshore macrofaunal communities exhibited strong seasonality, driven primarily by a few opportunistic and structurally dominant taxa. Taxon richness and abundance were not evenly distributed throughout the year; rather, they followed pronounced seasonal patterns determined by environmental conditions and the life-history strategies of the taxa.

## 4. Discussion

The Black Sea, a semi-enclosed inland sea, harbors a permanent anoxic layer starting from depths of 150–200 m. This condition, driven by the presence of hydrogen sulfide (H_2_S), limits the development of deep-sea benthic fauna [40,41]. Despite this limitation, the phylum Mollusca—particularly the classes Bivalvia and Gastropoda—plays a significant role within the region’s macroinvertebrate communities, ranking second in abundance after Arthropoda and first in terms of taxon richness [42,43,44].

However, studies on the molluscan taxa of the Black Sea along the Türkiye coast are scarce and scattered. Much of the existing literature is outdated or focused on the northern coasts of the basin (Russia, Romania, Ukraine) [3,42]. Bulgaria was among the pioneers of industrial mussel (*M. galloprovincialis*) cultivation in the Black Sea, with an annual market supply of approximately 150 tonnes prior to 1989 [45]. Surveys along the Türkiye coastline are mostly based on historical records and often lack contemporary ecological context [26,28,42,45]. Over the past fifteen years, *M. galloprovincialis* has emerged as one of the most economically valuable shellfish species in the Eastern Black Sea region, and several studies have been conducted on its cultivation [26,28,42,46].

In this context, our study fills a critical gap by providing up-to-date data on molluscan fauna through systematic sampling in offshore mussel aquaculture areas. In particular, the dominance of taxa such as *M. galloprovincialis*, *B. reticulatum*, and *A. inaequivalvis* in artificial habitats aligns with previously reported distribution patterns along the Black Sea coasts [45,47]. Among these, *A. inaequivalvis*, as an invasive taxon, can significantly influence local benthic community dynamics, a phenomenon also emphasized by [3].

Redundancy analyses revealed that environmental variables such as dissolved oxygen, pH, and substrate type are primary determinants of molluscan distribution [13,16]. These findings support the high environmental sensitivity of molluscan taxa and their potential use as indicators of habitat quality.

Beyond compositional aspects, taxa such as *M. galloprovincialis* contribute functionally by enhancing habitat complexity, stabilizing sediments, and filtering the water column [27]. However, the spread of an invasive taxon like *A. inaequivalvis* requires monitoring due to potential long-term impacts on aquaculture systems.

Studies on amphipods in the Black Sea indicate that some of them—particularly detritivores and epibenthic particle feeders—can reach high densities in organically enriched areas [48,49].

### 4.1. Abundance and Dominance Patterns of Macroinvertebrates

Reference [3] demonstrated that mussel aquaculture does not negatively affect benthic fish and macroinvertebrates. Considering that for a taxon such as the flounder, which lives in close association with bottom sediments and is sensitive to pollutants, a reduction in its abundance would be expected if aquaculture operations significantly contributed to pollution [50]. However, the abundance of a commercially important taxon such as *Pseudopleuronectes americanus* was found to be similar whether directly under mussel lines or at distant reference sites. Interestingly, both of these noted taxa were reported at lower densities in seagrass beds. The lower occurrence of *P. americanus* in seagrass habitats may be attributed to the structural complexity and competitive interactions within these systems, whereas mussel longline farms provide a more neutral and opportunistic habitat that aligns with the species’ preference for soft-bottom substrates and enhanced prey availability [50].

Previous studies have emphasized that the effects of artificial structures on sensitive ecosystems such as lagoons, bays, and estuaries depend on location and natural habitat type. Mussel farms are often established on sandy bottoms, where the addition of hard structures may prevent the full preservation of natural benthic community structures [45]. Cultivated mussel beds, however, have been found to support increased numbers of amphipod crustaceans. Reference [49] highlighted that crustaceans represented the dominant group within mussel aquaculture communities. Although species composition shifted on a monthly basis, *S. monoculoides*, *Melita palmata*, *J. marmorata*, and *Ericthonius brasiliensis* consistently accounted for the highest relative abundances [49].

In this study, *J. marmorata* (71%) was identified as the dominant taxon in offshore mussel aquaculture systems in the Black Sea, using mussel beds as shelter and food sources [51,52]. Similarly, *S. monoculoides* plays a significant role in supporting the diversity of amphipod taxa on biogenic substrates [53]. The dominance of *J. marmorata* in mussel aquaculture systems can be attributed to its ability to rapidly colonize artificial substrates, tube-building behavior, tolerance to organic enrichment, high reproductive potential, and competitive advantage, which collectively explain its prevalence in terms of both abundance and biomass in these habitats.

Macroinvertebrate abundance peaked in autumn, while taxon richness reached its highest level in summer. This pattern can be explained by the accumulation of organic matter in autumn supporting heterotrophic taxa [54,55], while more stable environmental conditions (temperature, oxygen, pH) in summer promote a higher diversity of taxa [54]. High Shannon–Wiener (~0.72) and Simpson (~0.42–0.43) values during summer indicate that environmental stability supports taxon diversity. Conversely, low Shannon–Wiener (~0.60) and Simpson (~0.37) values in winter suggest environmental stress linked to lower temperature and dissolved oxygen [56].

These ecological patterns were further confirmed through multivariate analyses. NMDS revealed clear seasonal differences in macroinvertebrate composition, with summer samples distinctly clustering due to higher taxon diversity and evenness. Notably, non-dominant taxa were more prevalent in summer, contributing to the compositional diversity of taxa. The PCA biplot showed that summer samples were strongly associated with temperature and salinity, whereas winter samples aligned with dissolved oxygen and pH, indicating lower taxon diversity and higher individual abundance. This supports the link between autumn abundance peaks and organic matter accumulation as well as the link between summer richness peaks and environmental stability.

The high values observed in winter and summer from Pielou’s Evenness Diversity Index suggest that dominant taxa (*J. marmorata* and *S. monoculoides*) possess broad environmental tolerance. The repeated appearance of these taxa in seasonal NMDS and PCA clusters highlights their potential as bioindicator taxa for ecosystem monitoring. Previous studies have also emphasized their tolerance to environmental variability in the Black Sea benthic systems [45,54].

Offshore mussel aquaculture systems function as artificial reef-like habitats, enhancing diversity. However, these benefits can only be sustained under appropriate environmental conditions and management practices. Low diversity and evenness values (S1 and S4 sampling periods) may indicate habitat degradation or stress. In this context, macrozoobenthic communities are used as biological quality elements in environmental monitoring frameworks such as the EU Water Framework Directive [15,16].

The observed seasonal patterns of higher abundance in autumn and higher taxon richness in summer demonstrate that these artificial habitats support diversity and functional cycles year-round.

### 4.2. Environmental Drivers of Community Structure

Among the environmental variables analyzed, pH and temperature emerged as the most influential factors structuring the macroinvertebrate community. RDA results demonstrated that pH primarily influenced sensitive taxa, including *H. crassipes*, *D. leucolena*, and *C. sinopae*, suggesting that fluctuations in alkalinity may directly regulate their settlement and survival [49]. In contrast, temperature strongly shaped the dynamics of dominant amphipods such as *J. marmorata* and *S. monoculoides*, which proliferated during warmer periods and exhibited wide ecological tolerance [49,57,58]. Opportunistic taxa, including *N. zonata*, were more closely related to dissolved oxygen, reaching higher abundances under winter conditions [59,60]. Rare taxa (*R. venosa*, *P. dumerilii*, *S. lactea*) did not show strong environmental associations, reflecting episodic recruitment events rather than consistent environmental filtering [59].

The seasonal variations in temperature produced the clearest community shifts, separating summer assemblages characterized by amphipod dominance from winter communities influenced by polychaetes and oxygen-rich conditions [61,62]. pH and salinity acted as secondary but significant gradients, differentiating sensitive taxa with narrower ecological requirements from more eurytopic taxa [48,63]. NMDS analysis further confirmed this structuring, indicating compositional differences between summer and winter assemblages (Bray–Curtis dissimilarity ≈ 0.50) [64,65]. Collectively, these results emphasize that pH regulates sensitive taxa, temperature drives dominant taxa, and oxygen shapes opportunistic taxa, revealing a strong seasonal turnover in community composition despite relatively stable diversity indices [56,61,66].

### 4.3. Management Implications for Mussel Aquaculture

While mussel aquaculture creates artificial habitats that enhance local diversity, it also introduces challenges such as space and resource competition with taxa, such as *Balanus improvisus* [64]. This issue becomes particularly prominent during larval settlement periods, potentially hindering mussel growth. Therefore, larval collector deployment should be carefully timed. To prevent settlement by *Balanus* larvae, which peak during July and August [3,64], collectors should be deployed in May, and surface cleaning strategies should be implemented to reduce colonization.

High macroinvertebrate biomass and diversity have been documented in tidal mussel aggregations [57,63]. Cultivated bivalves—particularly suspended mussel farming [55,57] as well as both surface and bottom oyster culture systems [64,67]—have been shown to support rich macroinvertebrate assemblages. The structures used in bivalve aquaculture provide favorable habitat conditions for macroinvertebrate taxon and are increasingly recognized as artificial reef systems that support not only macroinvertebrates but also larger marine fauna and fish [68,69]. Moreover, macroinvertebrate communities associated with suspended bivalve farming are generally considered a functional component of the benthic environment. Therefore, integrating data from both sediment-dwelling macroinvertebrates and those associated with mussel socks can yield a more comprehensive understanding of the effects of bivalve aquaculture on benthic ecosystems [59].

Although the current study recorded *R. venosa* at low densities, its known predatory behavior on mussels [59,70] suggests potential long-term effects on both mussel populations and associated macroinvertebrate communities. Future studies should incorporate targeted assessments of predator–prey interactions between *R. venosa*, *M. galloprovincialis*, and dominant amphipods (e.g., *J. marmorata*), especially given the ecological and economic importance of mussel aquaculture in the Black Sea.

In shellfish aquaculture, ecological carrying capacity is mostly considered in terms of “negative” impacts, typically associated with issues such as water quality degradation, sediment accumulation beneath farms, benthic enrichment, and effects on surrounding habitats [70]. Conversely, there is limited research exploring aquaculture sites as potential novel habitats that could enhance the abundance and productivity of marine organisms [60,62,71,72]. In addition, macroinvertebrate-based bioindicator programs should be integrated into aquaculture monitoring strategies. Taxa with stenotopic traits can serve as early warning indicators of environmental stress. Understanding the ecological functions of dominant taxa can guide habitat specific management practices.

Overall, the findings underscore the need for adaptive management that considers both production goals and ecological sustainability. Mussel aquaculture systems should be evaluated not only in terms of yield but also as dynamic ecosystems supporting diverse and responsive benthic communities.

## 5. Conclusions

Invertebrates inhabiting mussel beds contribute to nutrient cycling and ecological balance by consuming plankton, detritus, and microbial matter. In the Black Sea, benthic invertebrates play a critical role in maintaining ecosystem health. Mussel beds dominated by *M. galloprovincialis* form structured habitats that provide shelter and feeding grounds. Amphipods, in particular, find refuge among mussel shells and feed on organic matter.

This study assessed the ecological roles and diversity contributions of macroinvertebrates associated with *M. galloprovincialis* cultivated in mussel longline systems in the central Black Sea. Over a one-year monitoring period, *J. marmorata* emerged as the dominant taxon. Taxon richness peaked in summer, whereas individual abundance was highest in autumn.

The findings demonstrate that mussel aquaculture systems function not only as production platforms but also as habitats that support local diversity. However, ecological challenges such as competition with *B. improvisus* can be mitigated through adjustments in timing and culture depth.

In conclusion, offshore mussel aquaculture systems act as artificial reef-like habitats, enhancing benthic diversity and contributing to ecosystem resilience. These systems are both productive and ecologically valuable habitats in terms of taxon richness and sensitivity to environmental gradients. Sustaining habitat-sensitive aquaculture strategies is crucial for maintaining ecological integrity and economic viability.

## Figures and Tables

**Table 1:** Dominant macroinvertebrate taxa ranked by mean relative abundance, with notes on their ecological roles.

Taxon	Mean Relative Abundance (%)	Ecological Role
*Jassa marmorata*	71%	Opportunist, habitat forming
*Stenothoe monoculoides*	28%	Detritivore, inhabits sticky substrates
*Nereis zonata*	0.37%	Omnivore, sediment bioturbator
*Nematoda* (general)	0.12%	Microscopic, sensitive to organic matter
*Hyale crassipes*	0.10%	Detritivore, shows seasonal abundance trend

**Table 2:** Taxa identified in the study. (SUM: Total number of individuals. % D: Dominant taxa by relative abundance).

	September-2023	October	November	December	January	February	March	April	May	June	July	August-2024	SUM	% D
	S1	S2	S3	S4	S5	S6	S7	S8	S9	S10	S11	S12		
**Cnidaria**														
*Diadumene* sp.	0	3	0	0	0	0	0	0	13	45	14	3	78	0.0782
**Nemertea**														
*Lineus* sp.	0	3	0	0	0	0	0	0	0	0	0	0	3	0.0030
**Nematoda**														
Nematoda (sp.)	0	15	0	0	0	0	0	0	68	0	0	42	125	0.1254
**Platyhelminthes**														
*Cryptocelis sinopae* Gammoudi, Bulnes and Kurt, 2021	0	0	0	0	0	0	0	0	11	5	3	0	19	0.0191
**Polychaeta**														
*Nereis zonata* Malmgren, 1867	6	2	57	16	47	0	20	13	53	41	12	100	367	0.3680
*Perinereis cultrifera* (Grube, 1840)	0	0	0	0	0	0	0	0	0	0	0	3	3	0.0030
*Platynereis dumerilii* (Audouin and Milne Edwards, 1833)	0	5	0	0	0	0	0	0	0	0	0	0	5	0.0050
*Polyophthalmus pictus* (Dujardin, 1839)	0	0	0	0	0	0	0	0	6	0	0	1	7	0.0070
*Sigambra tentaculata* (Treadwell, 1941)	0	0	0	0	0	0	0	0	0	2	0	15	17	0.0170
**Crustacea**														
*Balanus improvisus* (Darwin, 1854)	7	0	16	2	1	0	0	1	0	0	2	0	29	0.0291
*Hyale crassipes* (Heller, 1866)	0	0	0	0	10	30	2	0	30	0	10	20	102	0.1023
*Jassa marmorata* Holmes, 1905	5.800	13.250	8.160	5.530	2.100	4.000	6.000	10.000	6.600	2.500	2.024	5.200	71.164	**71.3645**
*Pachygrapsus marmoratus* (Fabricius, 1787)	6	0	2	0	0	0	4	0	0	0	0	0	12	0.0120
*Palaemon longirostris* H. Milne Edwards, 1837	0	0	0	0	0	0	0	0	0	4	0	0	4	0.0040
*Pectenogammarus olivii* (H. Milne Edwards, 1830)	0	0	0	0	0	4	0	0	4	0	0	5	13	0.0130
*Pilumnus hirtellus* (Linnaeus, 1761)	0	0	9	0	0	4	2	0	0	7	0	0	22	0.0221
*Pisidia longicornis* (Linnaeus, 1767)	0	0	4	0	4	5	1	0	0	1	0	1	16	0.0160
*Stenothoe monoculoides* (Montagu, 1813)	1.900	5.000	5.290	1.040	500	2.000	2.000	2.000	4.520	1.000	1.006	1470	27.726	**27.8041**
**Mollusca**														
*Rapana venosa* (Valenciennes, 1846)	0	0	2	0	0	0	1	0	0	0	0	0	3	0.0030
*Striarca lactea* (Linnaeus, 1758)	0	0	2	0	2	0	0	0	0	0	0	0	4	0.0040

## Data Availability

Data are contained within the article.
