# Correction: Çil, E.A. Seasonal Dynamics of Macroinvertebrate Communities in Offshore Mussel Aquaculture in the Southern Black Sea: Implications for Diversity. *Life* 2025, *15*, 1471

**DOI:** 10.3390/life16010130

**Published:** 2026-01-15

**Authors:** Eylem Aydemir Çil

**Affiliations:** Department of Environmental Engineering, Faculty of Engineering and Architecture, Sinop University, 57000 Sinop, Türkiye; eylemaydemir@sinop.edu.tr

**Figure/Table Legend**

There is no error in the legend. However, in the original publication [1], the uncorrected versions of Figure 3 and Table 2 were inadvertently used instead of their corrected versions.

**Figure 3:**
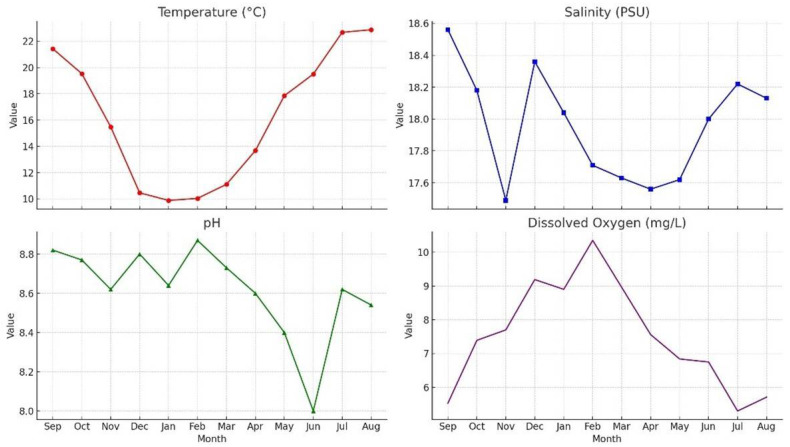
Monthly physicochemical parameter data.

**Table 2:** Taxa identified in the study. (SUM: Total number of individuals. % D: Dominant taxa by relative abundance).

	September-2023	October	November	December	January	February	March	April	May	June	July	August-2024	SUM	% D
	S1	S2	S3	S4	S5	S6	S7	S8	S9	S10	S11	S12		
**Cnidaria**														
*Diadumene* sp.	0	3	0	0	0	0	0	0	13	45	14	3	78	0.0782
**Nemertea**														
*Lineus* sp.	0	3	0	0	0	0	0	0	0	0	0	0	3	0.0030
**Nematoda**														
Nematoda (sp.)	0	15	0	0	0	0	0	0	68	0	0	42	125	0.1254
**Platyhelminthes**														
*Cryptocelis sinopae* Gammoudi, Bulnes and Kurt, 2021	0	0	0	0	0	0	0	0	11	5	3	0	19	0.0191
**Polychaeta**														
*Nereis zonata* Malmgren, 1867	6	2	57	16	47	0	20	13	53	41	12	100	367	0.3680
*Perinereis cultrifera* (Grube, 1840)	0	0	0	0	0	0	0	0	0	0	0	3	3	0.0030
*Platynereis dumerilii* (Audouin and Milne Edwards, 1833)	0	5	0	0	0	0	0	0	0	0	0	0	5	0.0050
*Polyophthalmus pictus* (Dujardin, 1839)	0	0	0	0	0	0	0	0	6	0	0	1	7	0.0070
*Sigambra tentaculata* (Treadwell, 1941)	0	0	0	0	0	0	0	0	0	2	0	15	17	0.0170
**Crustacea**														
*Balanus improvisus* (Darwin, 1854)	7	0	16	2	1	0	0	1	0	0	2	0	29	0.0291
*Hyale crassipes* (Heller, 1866)	0	0	0	0	10	30	2	0	30	0	10	20	102	0.1023
*Jassa marmorata* Holmes, 1905	5.800	13.250	8.160	5.530	2.100	4.000	6.000	10.000	6.600	2.500	2.024	5.200	71.164	**71.3645**
*Pachygrapsus marmoratus* (Fabricius, 1787)	6	0	2	0	0	0	4	0	0	0	0	0	12	0.0120
*Palaemon longirostris* H. Milne Edwards, 1837	0	0	0	0	0	0	0	0	0	4	0	0	4	0.0040
*Pectenogammarus olivii* (H. Milne Edwards, 1830)	0	0	0	0	0	4	0	0	4	0	0	5	13	0.0130
*Pilumnus hirtellus* (Linnaeus, 1761)	0	0	9	0	0	4	2	0	0	7	0	0	22	0.0221
*Pisidia longicornis* (Linnaeus, 1767)	0	0	4	0	4	5	1	0	0	1	0	1	16	0.0160
*Stenothoe monoculoides* (Montagu, 1813)	1.900	5.000	5.290	1.040	500	2.000	2.000	2.000	4.520	1.000	1.006	1470	27.726	**27.8041**
**Mollusca**														
*Rapana venosa* (Valenciennes, 1846)	0	0	2	0	0	0	1	0	0	0	0	0	3	0.0030
*Striarca lactea* (Linnaeus, 1758)	0	0	2	0	2	0	0	0	0	0	0	0	4	0.0040

**Error in Figure 3**

In Figure 3, the term “Dissolved Oxygen (g/kg)” was incorrectly used; in the corrected version it has been replaced with “Dissolved Oxygen (mg/L)”. The same correction (g/kg → mg/L) has also been made in the main text on Section 3.1 paragraph 5.

**Error in Table 2**

In Table 2, the order of the taxa had been modified according to the first reviewer’s recommendation; however, the incorrect (uncorrected) version of the table was mistakenly uploaded. It has now been replaced with the corrected version. Only the taxonomic order has been changed—the content and data remain the same.

**Missing Citation**

Only the reference list was corrected. Some citation errors and misspellings remained unedited prior to the reviewer suggestions, and these have now been carefully revised. No new citations were added.

**Text Correction**

The corrections made were on Section 3.1 paragraph 5 and Section 3.3 paragraph 5, 7, 8 and 9, where the expression “Dissolved Oxygen (g/kg)” was replaced with “mg/L” and the “Figure 5” should be “Figure 6” which was incorrectly numbered. The original “Figure 6” should be corrected as “Figure 7” and original “Figure 7” corrected as “Figure 6” because the figures need to appear after the first citation.

Dissolved Oxygen (DO): Dissolved oxygen levels peaked in February at 10.35 mg/L and reached their lowest level in July at 5.30 mg/L. This pattern corresponds to the reduced solubility of oxygen at higher temperatures and increased biological oxygen demand during the warmer months.

A pronounced increase in both taxon richness and individual abundance was observed during summer and early autumn (June–September). This seasonal peak coincides with periods of higher temperatures and reduced hydrodynamic impact, indicating enhanced macrofaunal productivity. The consistent dominance of *J. marmorata* during these months reflects the taxon’s rapid colonization ability and competitive advantage on artificial structures. In contrast, the lower and more stable abundances recorded in winter and spring are consistent with suppressed benthic activity under low temperatures and elevated oxygen conditions (Figure 6).

Figure 6 has been moved to after its first mention.

While the dominant amphipods *J. marmorata* and *S. monoculoides* largely shaped the overall temporal trends, several low-abundance taxa such as *Balanus improvisus*, *C. sinopae*, *H. crassipes*, and *N. zonata* contributed to short-term peaks in richness during specific sampling periods (e.g., S4, S9) (Table 2). Although these episodic fluctuations had minimal influence on total abundance, they underscore the ecological variability of the community and the occurrence of sporadic settlement events (Figure 7).

The RDA biplot illustrated the relationships between taxa and environmental variables (pH, dissolved oxygen, temperature, and salinity) (Figure 6). These findings are of considerable importance for understanding the influence of environmental variables on taxon distribution and habitat preferences.

The results of RDA indicated that pH and temperature were the most influential physicochemical factors shaping taxa distribution. pH showed a strong relationship to sensitive taxa such as *H. crassipes, D. leucolena*, and *C. sinopae*, suggesting that even small fluctuations in alkalinity may regulate their settlement and persistence. In contrast, the dominant amphipods *J. marmorata* and *S. monoculoides* were primarily aligned with temperature along the first axis, reflecting their seasonal proliferation during warmer months. The opportunistic polychaete *N. zonata* was more closely associated with oxygen, showing higher abundances during winter. Rare or episodic taxa (*Rapana venosa*, *Platynereis dumerilii*, *Striarca lactea*) exhibited no strong correlation with environmental gradients, instead reflecting sporadic recruitment events. Overall, the results demonstrate a clear seasonal structuring of the community, with pH influencing sensitive taxa, temperature driving dominant taxa, and oxygen regulating opportunistic taxa (Figure 6). This pattern indicates that community structure beneath mussel aquaculture is strongly influenced by seasonal hydrographic variations.

**References**

Author revised some of the references, they are listed as follows:

4. Aral, O. Growth of the Mediterranean mussel (*Mytilus galloprovincialis* lam., 1819) on ropers in the Black Sea, Turkey. *Turk. J. Vet. Anim. Sci.* **1999**, *23*, 183–190.
5. Ivanov, V.N.; Bulatov, K.V. Population genetic investigations of Black Sea *Mytilus galloprovincialis* Lam. *Hydrores* **1990**, *7*, 106–111.
6. Aydemir-Çil, E.; Birinci-Özdemir, Z.; Özdemir, S. First find of the starfish, *Asterias rubens* Linnaeus, 1758, off the Anatolian coast of the Black Sea (Sinop). *Mar. Biol. J.* **2023**, *8*, 97–101. https://doi.org/10.21072/mbj.2023.08.3.07.
26. Acarlı, S.; Vural, P.; Yıldız, H. An and suitability for human consumption of Mediterranean mussels (*Mytilus galloprovincialis* Lamarck, 1819) from the Yalova coast of the Marmara Sea. *Memba Kastamonu Üniv. Su Ürünleri Fak. Derg.* **2023**, *9*, 12–24.
27. Turan, H.; Sönmez, G.; Çelik, M.Y.; Yalçın, M.; Kaya, Y. Effects of different salting process on the storage quality of Mediterranean mussel (*Mytilus galloprovincialis* L. 1819). *J. Muscle Foods* **2007**, *18*, 380–390. https://doi.org/10.1111/j.1745-4573.2007.00093.x.
28. Mititelu, M.; Neacșu, S.M.; Oprea, E.; Dumitrescu, D.E.; Nedelescu, M.; Drăgănescu, D.; Nicolescu, T.O.; Roșca, A.C.; Ghica, M. Black Sea mussels qualitative and quantitative chemical analysis: Nutritional benefits and possible risks through consumption. *Nutrients* **2022**, *14*, 964. https://doi.org/10.3390/nu14050964.
29. Çınar, M.E.; Açık, Ş.; Kurt, G.; Erdoğan Dereli, D. Diversity of Annelida from the coasts of Türkiye. *Turk. J. Zool.* **2024**, *48*, 413–445. https://doi.org/10.55730/1300-0179.3193.
31. Fauchald, K. The Polychaete Worms. Definitions and Keys to the Orders, Families and Genera. In *Natural History Museum of Los Angeles County Science Series*; Natural History Museum of Los Angeles County: Los Angeles, CA, USA, 1977; Volume 28, pp. 1–188.
32. Hutchings, P.; Murray, A. *Taxonomy of Polychaetes from the Hawkesbury River and the Southern Estuaries of New South Wales, Australia (Vol. 3)*; Australian Museum: Sydney, Australia, 1984; Volume 3, pp. 1–118. https://doi.org/10.3853/j.0812-7387.3.1984.101.
44. Anistratenko, V.V.; Anistratenko, O.Y. Fauna Ukraine: In 40 vol. Vol. 29: Mollusca. Fasc. 1. B. 1: Class Polyplacophora or Chitons, Class Gastropoda—Cyclobranchia, Scutibranchia and Pectinibranchia (part). *Kiev Veles*. **2001**, 1–240.
50. Brander, K.; Cochrane, K.; Barange, M.; Soto, D. Climate change implications for fisheries and aquaculture. *Clim. Change Impacts Fish. Aquac. A Glob. Anal.* **2017**, *1*, 45–62. https://doi.org/10.1002/9781119154051.ch3.
53. Aslan, H.; İşmen, P. Peracarid crusteceans species from upper infralittoral rocky shores of Gokceada Island (Aegean Sea). *Çanakkale Onsekiz Mart Univ. J. Mar. Sci. Fish.* **2019**, *2*, 109–119.
55. Filgueira, R.; Grant, J.; Petersen, J.K. Identifying the optimal depth for mussel suspended culture in shallow and turbid environments. *J. Sea Res.* **2018**, *132*, 15–23. https://doi.org/10.1016/j.seares.2017.11.006.
56. Roman, M.R.; Altieri, A.H.; Breitburg, D.; Ferrer, E.M.; Gallo, N.D.; Ito, S.I.; Limburg, K.; Rose, K.; Yasuhara, M.; Levin, L.A. Reviews and syntheses: Biological indicators of low-oxygen stress in marine water-breathing animals. *Biogeosciences* **2024**, *21*, 4975–5004. https://doi.org/10.5194/egusphere-2024-616.
61. Govorin, I.A. The predatory marine gastropod *Rapana venosa* (Valenciennes, 1846) in Northwestern Black Sea: Morphometric variations, imposex appearance and biphallia phenomenon. In *Molluscs*; IntechOpen: London, UK, 2019; pp. 31–45. https://doi.org/10.5772/intechopen.81209.
64. Karayücel, S.; Çelik, M.Y.; Karayücel, İ.; Erik, G. Growth and production of raft cultivated Mediterranean mussel (*Mytilus galloprovincialis* Lamarck, 1819) in Sinop, Black Sea. *Turk. J. Fish. Aquat. Sci.* **2010**, *10*. https://doi.org/10.4194/trjfas.2010.0102.

The authors state that the scientific conclusions are unaffected. This correction was approved by the Academic Editor. The original publication has also been updated.
